# Pyrolytic carbon resonators for micromechanical thermal analysis

**DOI:** 10.1038/s41378-019-0094-x

**Published:** 2019-10-21

**Authors:** Long Quang Nguyen, Peter Emil Larsen, Tom Larsen, Sanjukta Bose Goswami, Luis Guillermo Villanueva, Anja Boisen, Stephan Sylvest Keller

**Affiliations:** 1 DNRF and Villum Fonden Center for Intelligent Drug Delivery and Sensing Using Microcontainers and Nanomechanics, IDUN, Technical University of Denmark, 2800 Kgs Lyngby, Denmark; 2 DTU Nanolab, Technical University of Denmark, 2800 Kgs Lyngby, Denmark; 3 DTU Health Tech, Technical University of Denmark, 2800 Kgs Lyngby, Denmark; 4 Advanced NEMS Laboratory, Ecole Polytechnique Fédérale de Lausanne, 1015 Lausanne, Switzerland

**Keywords:** Electrical and electronic engineering, Sensors, NEMS

## Abstract

Thermal analysis is essential for the characterization of polymers and drugs. However, the currently established methods require a large amount of sample. Here, we present pyrolytic carbon resonators as promising tools for micromechanical thermal analysis (MTA) of nanograms of polymers. Doubly clamped pre-stressed beams with a resonance frequency of 233 ± 4 kHz and a quality factor (Q factor) of 800 ± 200 were fabricated. Optimization of the electrical conductivity of the pyrolytic carbon allowed us to explore resistive heating for integrated temperature control. MTA was achieved by monitoring the resonance frequency and quality factor of the carbon resonators with and without a deposited sample as a function of temperature. To prove the potential of pyrolytic carbon resonators as thermal analysis tools, the glass transition temperature (*T*_g_) of semicrystalline poly(L-lactic acid) (PLLA) and the melting temperature (*T*_m_) of poly(caprolactone) (PCL) were determined. The results show that the *T*_g_ of PLLA and *T*_m_ of PCL are 61.0 ± 0.8 °C and 60.0 ± 1.0 °C, respectively, which are in excellent agreement with the values measured by differential scanning calorimetry (DSC).

## Micromechanical analysis: pyrolytic carbon resonators effective in micromechanical thermal analysis

Pyrolytic carbon is a material similar to graphite, and resonators (devices that oscillate with greater amplitude at certain frequencies) made from pyrolytic carbon show considerable promise in the micromechanical thermal analysis (MTA) of nanogram samples of polymers. Thermal analysis is essential for characterizing polymers and drugs, but conventional analytic techniques typically demand several milligrams of the sample, which may be unavailable or excessively expensive. A team headed by Long Quang Nguyen at the Technical University of Denmark was able to develop a simple fabrication process to produce electrically conductive pyrolytic carbon resonators for MTA of polymer samples at the nanogram level. The authors believe that pyrolytic carbon resonators have great potential in MTA; toward expanding those possibilities, they aim in future to examine whether they can enhance the thermal range of the resonators.

## Graphical abstract

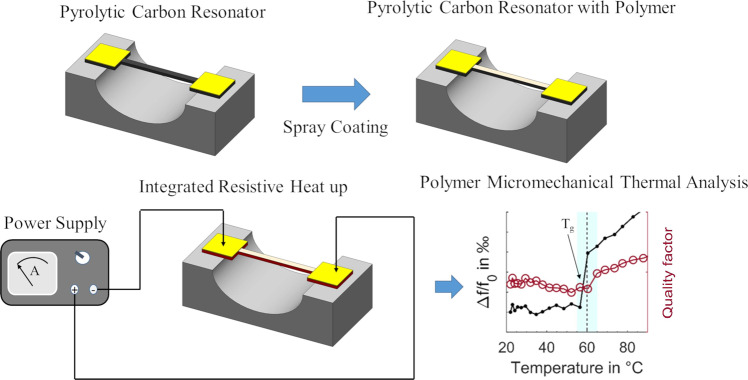

## Introduction

Thermal analysis refers to a number of different techniques involving temperature control to study the material properties of a sample. These techniques include thermogravimetry (TGA), dynamic mechanical analysis (DMA), differential thermogravimetry (DTG), differential scanning calorimetry (DSC), differential thermal analysis (DTA) and temperature-programmed desorption (TPD). The information obtained through thermal analysis provides an understanding of thermal properties, such as the glass transition temperature *T*_g_ or the melting temperature *T*_m_, of a variety of materials, such as polymers^1,2^, pharmaceutical compounds^3^ or proteins^4^. However, these techniques typically require a few milligrams of sample, which is not always available or only available at high costs. To address this limitation, thermal analysis using microelectromechanical system (MEMS) structures, such as freestanding beams or membranes, has been introduced. The principle of this method is based on tracking the resonance frequency shift and Q factor of the micromechanical resonators during controlled temperature ramps, followed by correlating the resonant behavior with changes in the thermal properties of the analyzed sample. Resonating sensors, such as quartz microresonators^5,6^, micromechanical cantilevers^5,7^, and micromechanical string resonators^8,9^, have been employed for micromechanical thermal analysis (MTA) of nanogram samples due to the small size of the sensors. The excellent performance of resonating microsensors, specifically the high sensitivity and the short thermal response time, has been explored for various thermal analysis applications^10–16^. Furthermore, micromechanical resonators are suitable for integration with Si-based microelectronics.

To date, in order to use micromechanical resonators for thermal analysis, an external heat source, such as a Peltier element^8^, a metal ceramic heater^9^ or a holder with temperature control^17^, has been used. This approach results in a relatively complicated setup for controlling the temperature during MTA and a slow thermal response due to the large thermal mass of the measurement system. Therefore, direct integration of heating and temperature control into the resonators would simplify the setup and significantly reduce the time constant during the measurements. In several studies, microheaters have been integrated with quartz crystal microbalance (QCM) sensors for measuring particulate matter^18^ or the viscosity of materials^19^. Furthermore, microheaters have also been integrated with suspended membranes for gas sensing^20^ or doubly clamped beams for calorimetry^21^. To integrate these microheaters, metals, such as Au or Pt, have been fabricated as patterned resistor structures on the sensors. However, this approach requires more steps in the fabrication process, which is time consuming and expensive.

In the past decade, carbon MEMS (C-MEMS) has been established as a method to fabricate carbon microstructures. Basically, in the C-MEMS process, a photoresist precursor structure is pyrolysed at high temperature in an inert atmosphere and converted into pyrolytic carbon^22,23^. To date, pyrolytic carbon has been successfully applied in electrochemistry. By using pyrolytic carbon as an electrode material, many electrochemical^24,25^ or bionanoelectronics^26^ applications have been developed. Furthermore, 3D pyrolytic carbon structures have been used for improving the sensor performance^27,28^ or developing microbatteries^29,30^. Recently, pyrolytic carbon string resonators have been successfully fabricated with a simple and fast process^31,32^. The optimized micromechanical string resonators display excellent resonant behavior, which has been suitable for applications such as mass sensing and nanomechanical infrared (NAM-IR) spectroscopy^33^. Further optimization of the process resulted in doubly clamped pyrolytic carbon resonators that are electrically conductive, and this property has been explored for the electrodeposition of nanoparticles on suspended structures^34^. Recently, Salazar et al. fabricated suspended pyrolytic carbon nanowires by electromechanical spinning and demonstrated resistive heating that could locally reach temperatures >2000 °C^35^.

In this study, we applied electrically conductive pyrolytic carbon resonators as tools for the thermal analysis of nanogram samples of polymers. For this purpose, doubly clamped pre-stressed beam structures served as both micromechanical sensors and resistive elements for integrated heating. First, the electrical resistance and the temperature coefficient of resistance of the pyrolytic carbon were characterized. Next, the resonance frequencies before and after polymer deposition by spray coating were used to estimate the deposited mass. Finally, the resonance frequency and Q factor of the carbon resonators were measured during a controlled increase of the temperature by resistive heating. The changes in the resonant behavior allowed us to identify the *T*_g_ of semicrystalline poly(lactic acid) (PLLA) and *T*_m_ of poly(caprolactone) (PCL).

## Results and discussion

### Pyrolytic carbon resonators

**Fig. 1:**
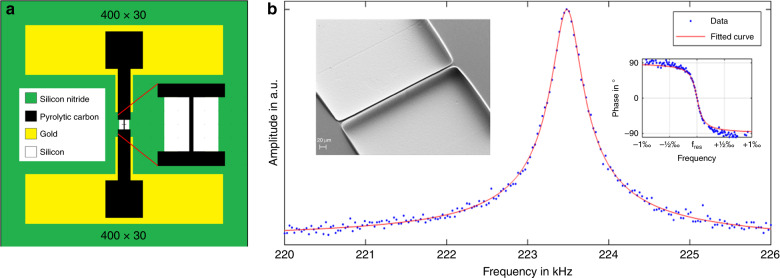
Pyrolytic carbon resonator. **a** Design of the doubly clamped pyrolytic carbon resonator; **b** SEM image (inset) and resonance frequency peak of the first mode

The C-MEMS method was applied for the fabrication of doubly clamped pyrolytic carbon resonators following a process similar to that previously described^34^. The final design of the devices is shown in Fig. 1a. The negative photoresist SU-8 was patterned by standard UV photolithography to define resonators and contact electrodes on a SiN insulation layer. This step was followed by release of the doubly clamped SU-8 beams using isotropic etching and pyrolysis. Finally, Au electrode contacts were deposited by e-beam metal evaporation through a shadow mask. Figure 1b shows an SEM image (inset) and the first mode resonance frequency peak of a pyrolytic carbon resonator with a nominal length of 400 µm, a nominal width of 30 µm and a thickness of 0.6 µm. The resonance frequencies of the pyrolytic carbon resonator were 233 ± 4 kHz for the first mode and 550 ± 7 kHz for the second mode, which are similar to the values previously reported for devices fabricated under the same pyrolysis conditions^34^. For ideal string-like resonators, the ratio between the frequencies of the second and first modes is 2, whereas for completely stress-free doubly clamped beams, it is 2.76^36^. In this study, this ratio was 2.36, which means that the resonators were not perfect strings but merely pre-stressed doubly clamped beams. Nevertheless, the fabricated pyrolytic carbon resonators should be suitable for thermal analysis of polymers.

### Electrical resistance

**Fig. 2:**
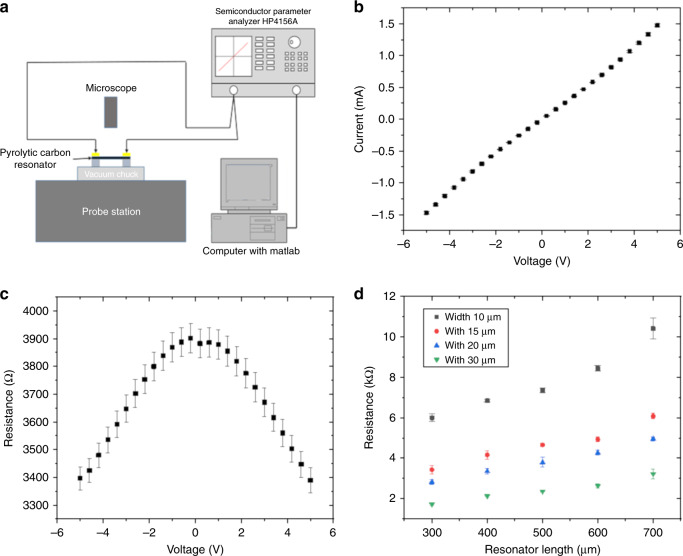
Electrical characterization of pyrolytic carbon resonators. **a** probe station setup; **b** I–V curve and **c** calculated electrical resistance of pyrolytic carbon resonators with a length of 400 µm and a width of 30 µm pyrolysed at 900 °C; **d** electrical resistances of pyrolytic carbon resonators with various dimensions pyrolysed at 900 °C

The ultimate goal of this study was to apply pyrolytic carbon resonators with integrated temperature control as tools for thermal analysis. To induce resistive heating of the suspended structures by an applied current, the electrical resistance of the resonators had to be in the range of kΩ. A larger resistance would require high voltages to dissipate the high power, whereas a lower resistance would require high currents to obtain sufficient resistive heating to increase the temperature. The pyrolysis temperature was previously identified as the most important parameter influencing the resistivity of pyrolytic carbon^37^. To adjust the resistance, pyrolytic carbon resonators with pyrolysis temperatures of 700 °C, 900 °C, and 1100 °C were fabricated. The electrical resistances of the pyrolytic carbon resonators were measured using a probe station (Fig. 2a). Figures 2b, c show the current-voltage (I–V) curves and the calculated resistance of the pyrolytic carbon resonators with a nominal length of 400 µm and a nominal width of 30 µm obtained at 900 °C. The results show that the electrical resistance of the resonators displayed an ohmic behavior for small applied potentials in the range of −2 V to 2 V. The resistance was calculated as the average value between −0.5 V and 0.5 V, giving a value of 3.9 ± 0.2 kΩ. For larger potentials, a decrease in the resistance was observed (Fig. 2c). This decrease was attributed to the resistive heating of the resonators due to the increasing current. Several resonator designs with different lengths and widths were fabricated for comparison. The electrical resistances of the pyrolytic carbon resonators with different lengths and widths were measured, and the results are shown in Fig. 2d. The results show a linear increase in the resistance with increasing length of the resonators, which confirmed the ohmic behavior of the pyrolytic carbon resistors. With the resistance and the dimensions of the resonators, the resistivity of pyrolytic carbon obtained at a pyrolysis temperature of 900 °C was calculated to be 16.2 ± 0.1 × 10^−3^ Ω·cm, which is comparable with values reported in another study^37^. At the lower pyrolysis temperature of 700 °C, the resistances were three orders of magnitude higher [Supporting Information S1]; therefore, these devices were not used in further experiments. For resonators pyrolysed at 1100 °C, the resistances were lower [Supporting Information S1]. However, for these structures, buckling due to compressive stress was observed^32^. In conclusion, the resonators fabricated at 900 °C had resistances in the kΩ range and were deemed suitable for resistive heating. For all further experiments, resonators with a nominal length of 400 µm and a nominal width of 30 µm were used.

### Resistive heating of pyrolytic carbon resonators

To determine the actual temperature of the pyrolytic carbon during resistive heating, the temperature coefficient of resistance (TCR) of pyrolytic carbon had to be measured. The TCR defines the change in resistance as a function of temperature and is calculated as:

1 $${\rm{TCR}} = \frac{{R_1 - R_0}}{{R_0(T_1 - T_0)}}$$

where *R*_0_ is the resistance at room temperature, *R*_1_ is the resistance at operating temperature, and *T*_0_ and *T*_1_ are room temperature and the operating temperature, respectively.

**Fig. 3:**
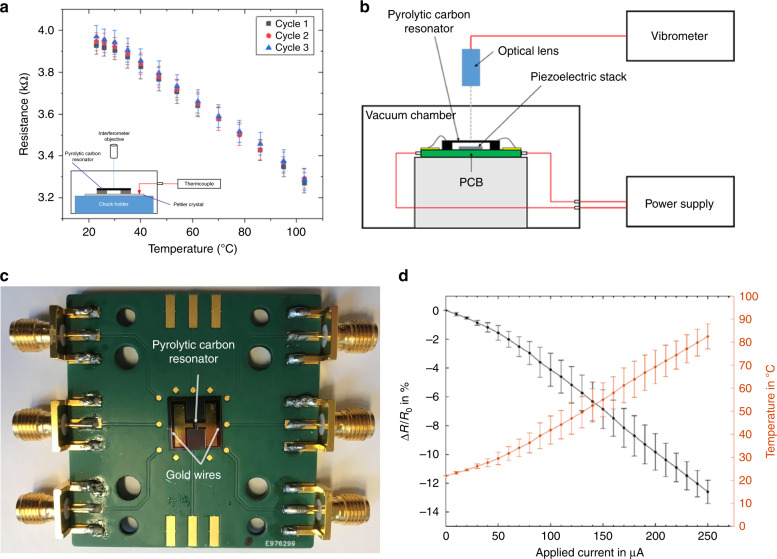
Integrated resistive heating characterization. **a** Resistance of pyrolytic carbon resonators as a function of temperature externally controlled by a Peltier element (inset); **b** experimental setup for integrated resistive heating of pyrolytic carbon resonators; **c** device mounted on PCB; **d** measured change in resistance due to resistive heating, and calculated temperature of pyrolytic carbon resonators

To determine the TCR of pyrolytic carbon, a Peltier element was used as the external heat source. The pyrolytic carbon resonators were heated up and cooled down for 3 cycles while the resistance was measured. Figure 3a shows that the resistance of the pyrolytic carbon resonators decreased with increasing temperature and that this behavior was repeatable during temperature cycling. The TCR is calculated as the average slope of the resistance value (ΔR/ΔT) in the linear interval. Based on Eq. (1), the TCR of pyrolytic carbon was calculated as −0.0017 ± 0.0003 ppm/K (*n* = 6). This negative TCR value of pyrolytic carbon is similar to the negative TCR values reported for graphite (−0.0005 ppm/K)^38^. A similar decrease in the resistance was observed under external heating of the string resonators based on the readout laser of a vibrometer operated at high power, confirming the negative TCR value for pyrolytic carbon [Supporting Information S2].

For the evaluation of integrated resistive heating, the pyrolytic carbon resonators were mounted on a printed circuit board (PCB), and a current was applied through the suspended structures under high vacuum (Fig. 3b, c). For initial temperature calibration, the actual temperature of the resonators was estimated using the TCR of pyrolytic carbon determined above. The voltage drop across the resonator was measured when applying the current, and the resistance of the carbon resonator was obtained using Ohm’s law. With the room temperature *T*_0_ and the calculated resistances *R*_0_ and *R*_1_, Eq. (1) was applied to determine the actual temperature *T*_1_ of the pyrolytic carbon resonators. Figure 3d shows the measured change in resistance as a function of the applied current and the resulting temperature calibration obtained using the TCR of pyrolytic carbon. When the applied current was increased, the resistance of the pyrolytic carbon resonators linearly decreased. As a consequence, the calculated temperature of the resonators increased with a linear dependence on the current, confirming resistive heating. This temperature calibration was used to determine the temperature of the polymer-coated resonators in MTA. Here, it is important to note that the temperature was approximated as an average value for the resonator. The effective temperature profile along the length of the doubly clamped beams should display a maximum value in the center and a lower temperature at the clamped ends.

### Deposition of polymer samples

**Fig. 4:**
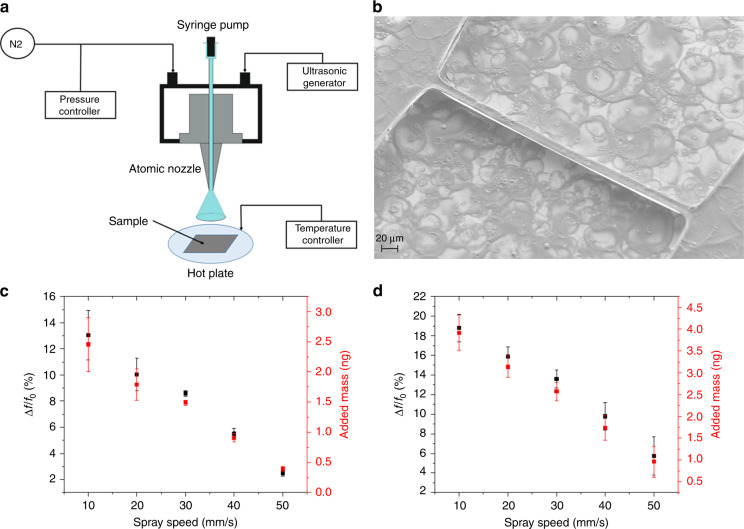
Polymer deposition and mass estimation. **a** Setup for ultrasonic spray coating of polymers on resonators; **b** SEM image of a carbon resonator after PLLA deposition; decrease in resonance frequency and estimated added mass for different spray speeds with **c** 1 spray coating pass and **d** 2 passes

Polymer samples were deposited onto pyrolytic carbon resonators using spray coating. Figure 4a shows an SEM image after successful deposition of PLLA on the surface of a carbon resonator. The polymer film was uniform on the top surface of the carbon resonator.

Experiments with different numbers of spray passes and different spray speeds were performed to compare the masses of the polymer added to the resonators. For each condition, the resonance frequency of the resonators was measured before and after the deposition to estimate the added mass based on the following equation^36^:

2 $$\Delta m = m_0\left( {\left( {\frac{{f_0}}{{f_0 - \Delta f}}} \right)^2 - 1} \right)$$

where *∆m* is the change in mass, *∆f* is the change in the resonance frequency, and *m*_*o*_ and *f*_*o*_ are the mass and resonance frequency of the resonator before deposition, respectively. Equation (2) is based on the approximation that the effective stiffness of the resonator is constant before and after deposition and that the deposited mass is distributed evenly over the length of the resonator. To calculate the added mass using Eq. (2), the density of the pyrolytic carbon had to be estimated. With the density measurement, the density of pyrolytic carbon was calculated as 1.36 ± 0.18 g/cm^3^, which is similar to values reported in another study (1.52 ± 0.06 g/cm^3^)^39^. The slight difference between the two values can be explained by the difference in pyrolysis process parameters. Figure 4b, c summarize the change in resonance frequency and the added mass for different numbers of spray passes and different spray speeds. For all the samples, <5 ng of PLLA was deposited. The results show linear decreases in the resonance frequency shift and the added mass when the spray speed was increased for both one and two spray passes. In both cases, less polymer was deposited at higher spray speeds, as would be expected. Meanwhile, when the number of spray passes was increased from one to two, more polymer was successfully deposited on the beams, leading to larger resonance frequency shifts, as well as more added mass. With increasing thickness of the deposited polymer layer, the assumption of constant resonator stiffness will increasingly lead to underestimation of the added mass.

### Micromechanical thermal analysis of polymers

**Fig. 5:**
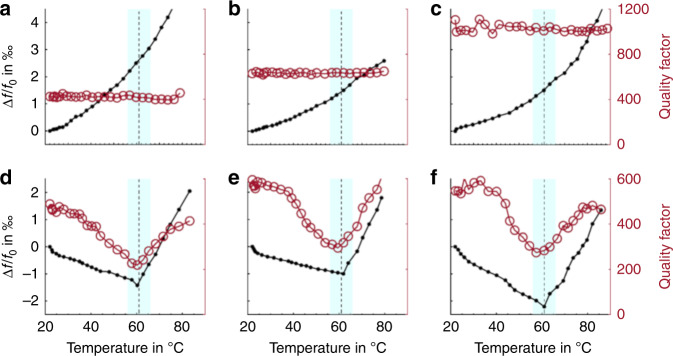
Micromechanical thermal analysis using pyrolytic carbon resonators. **a**–**c** before and **d**–**f** after poly(lactic acid) coating

The micromechanical thermal analysis of polymers in this study is based on resonance frequency measurements of pyrolytic carbon resonators during temperature ramping. Therefore, first, the resonant behavior of uncoated (blank) pyrolytic carbon resonators upon heating was investigated. The temperature of a resonator was increased by resistive heating while the resonance frequency and Q factor of the carbon resonator were recorded. Figure 5a–c show that when the temperature was increased, the resonance frequency increased. The resistive heating is limited to the pyrolytic carbon resonators. Therefore, this observation indicates an increase in the tensile stress in the doubly clamped beam due to the negative coefficient of thermal expansion (CTE) of the pyrolytic carbon. For temperatures <200 °C, negative values of CTE have frequently been reported for other carbon materials such as graphite or pyrolytic carbon deposited by chemical vapor deposition^40,41^. A similar increase in the resonance frequency was observed under external local heating of the string resonators based on the readout laser of a vibrometer operated at high power [Supporting Information S2], confirming the negative CTE for pyrolytic carbon. In terms of the Q factor, the value was stable when the temperature increased, indicating a negligible change in resonator damping.

For the micromechanical thermal analysis, PLLA was spray coated on pyrolytic carbon resonators (2 passes, 30 mm/s). The polymer-coated carbon resonators were heated by resistive heating under the same conditions as the blank resonators. Figure 5d–f show the thermal analysis of PLLA using three different pyrolytic carbon resonators. Two different temperature regimes were identified. In the first regime, a linear decrease in the resonance frequency was observed until the resonators reached a temperature of ~60 °C. After this point, in the second regime, the resonance frequency linearly increased. A similar behavior was observed for the Q factor of the resonators. The initial decrease in the resonance frequency was attributed to the thermal expansion of the PLLA with a positive CTE_PLLA_ = 58.4 ppm/K^8^, in contrast to the negative CTE of the pyrolytic carbon. The change in slope at approximately 60 °C is related to the glass transition temperature *T*_g_ of PLLA. During the glass-rubber phase transition, the Young’s modulus of PLLA considerably decreases^42^. Therefore, the mechanical properties of the resonator above *T*_g_ are dominated by the properties of the pyrolytic carbon. Hence, after passing *T*_g_, the resonance frequency increased as a function of temperature, as was observed for the blank carbon resonators. Based on the minimum value of the resonance frequency, *T*_g,PLLA_ was determined to be 61.0 ± 0.8 °C (*n* = 3). This temperature was comparable with the *T*_g,PLLA_ of 60.8 °C measured by DSC^43^. The gradual decrease and subsequent increase in the Q factor is caused by the gradual increase in molecular movement within the polymer, which results in a maximum of the viscous damping within the polymer at *T*_g_. Above this temperature, the mechanical strength of the polymer layer decreases, which reduces the damping contribution of the polymer layer, and the Q factor recovers.

**Fig. 6:**
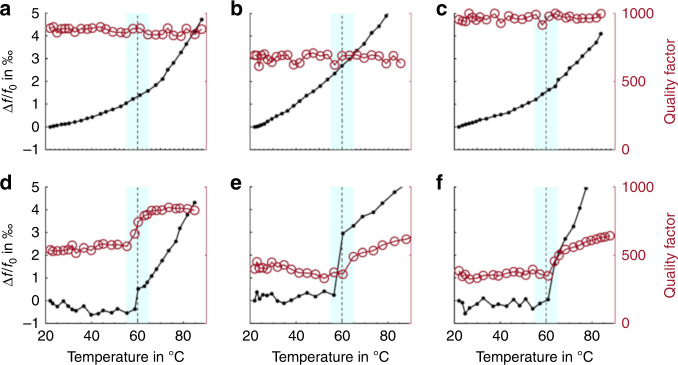
Micromechanical thermal analysis using pyrolytic carbon resonators. **a**–**c** before and **d**–**f** after poly(caprolactone) coating

For further investigation of the potential of pyrolytic carbon resonators as a tool for thermal analysis, PCL was deposited on pyrolytic carbon resonators by spray coating using the same parameters as for PLLA. Figure 6 shows the thermal analysis of PCL using resistive heating of three different pyrolytic carbon resonators. At low temperatures, the resonance frequency of the coated resonators remained relatively stable. However, above 60 °C, a step-shaped increase in the resonance frequency was observed. This change in resonant behavior was attributed to the melting of PCL at the melting temperature *T*_m,PCL_. At *T*_m_, a polymer changes from a semicrystalline state to an amorphous state, in principle losing all pre-orientation of the polymer chains. This phase change might result in a loss of structural integrity of the polymer and less damping of the resonator, leading to recovery of the Q factor. Furthermore, reorganization of mass on the surface of the resonators, e.g., due to polymer reflow, can lead to changes in the effective resonator mass, resulting in a sudden change in the resonance frequency^9^. Based on the change in slope in the frequency measurements, the melting temperature of PCL was determined to be *T*_m,PCL_ = 60.0 ± 1.1 °C, which is similar to the melting temperature of PCL reported in the literature (57.8 ± 4 °C)^44^.

## Conclusion

In this study, electrically conductive pyrolytic carbon resonators were developed and established as a tool for micromechanical thermal analysis. With a simple fabrication process, the pyrolytic carbon resonators show good resonant behavior with a resonance frequency of 233 kHz and Q factors between 600 and 1000 in vacuum. The carbon resonators are suitable for resistive heating using currents in the range of µA. The resistive heating allows for internal temperature control of the carbon resonators and the possibility to perform temperature ramping during resonance frequency measurements. Furthermore, the temperature coefficient of resistance of pyrolytic carbon is negative and estimated to be −0.0017 ± 0.0003 ppm/K. After temperature calibration, the sensors were used as a tool for thermal analysis of the glass transition temperature (*T*_g_) of PLLA and melting temperature (*T*_m_) of PCL. Compared to heating with an external Peltier element, the integrated resistive heating allows measurements immediately after increasing the temperature due to the low thermal mass of the system. The results of the micromechanical thermal analysis give *T*_g,PLLA_ = 61.0 ± 0.8 °C and *T*_m,PCL_ = 60.0 ± 1.1 °C, which are in excellent agreement with the literature. Furthermore, our results indicate a negative coefficient of thermal expansion (CTE) for pyrolytic carbon. In the future, we will explore whether the temperature range for thermal analysis with pyrolytic carbon resonators can be further increased. The results show the promising potential of conductive pyrolytic carbon resonators for micromechanical thermal analysis.

## Materials and methods

### Pyrolytic carbon resonator fabrication

The fabrication process of pyrolytic carbon resonators used in this study was previously described in detail^34^. Briefly, pyrolytic carbon resonators were fabricated using silicon nitride dry etching, SU-8 photolithography, isotropic silicon etching, pyrolysis and e-beam metal evaporation. However, in this work, the design of the resonators (Fig. 1a) was modified, and the thickness of the resonators was reduced to 0.6 µm. Pyrolytic carbon resonators with different lengths and widths were prepared for characterization of the electrical resistance.

### Electrical resistance measurement

The electrical resistances of the carbon resonators were measured by a probe station and a Semiconductor Parameter Analyzer HP4156A (Fig. 1b). The applied voltage was varied from −5 V to 5 V while the current was measured. The Semiconductor Parameter Analyzer HP4156A was controlled by MATLAB software to obtain the I–V curves and the resistance values between the two terminals.

### Polymer preparation and spray coating process

To investigate the potential of the pyrolytic carbon resonators as a tool for thermal analysis, two polymers were characterized, and 0.5 wt.% solutions of poly(L-lactic acid) (PLLA) (*M*_w_ 204,800 g/mol, NatureWorks PLA polymer 2003D) and poly(caprolactone) (PCL) (*M*_w_ 80,000 g/mol, Sigma Aldrich) with dichloromethane (DCM, Sigma Aldrich) as a solvent were prepared. The polymer solutions were spray coated on one side of the pyrolytic carbon resonators with an Exacta Coat Ultrasonic Spray System (Sonotek, USA) in a way similar to that previously described (Fig. 4a)^8^. The tip of the ultrasonic atomizer nozzle (Accumist, Sonotek, USA) was actuated at a frequency of 120 kHz with a generator power of 1.3 W, compressed nitrogen for shaping air at 0.04 kPa and a solution flow rate of 0.1 ml/min. In this work, the distance from the nozzle tip to the substrate was fixed at 4 cm, and the temperature was kept constant at room temperature. The translational movement speed of the spraying nozzle (10–50 mm/s) and the number of times the nozzle passed over the substrates (1 or 2 passes) were changed to deposit different masses of polymer on the resonators.

### Density measurement

For the estimation of the added mass of spray-coated polymers, the density and thickness of the pyrolytic carbon string resonator material had to be determined. For this purpose, unpatterned SU-8 photoresist films were processed on 4 inch Si wafers using parameters identical to those for the fabrication of the string resonators. The samples were weighed on a microbalance before spin-coating and after pyrolysis of the polymer films to obtain the weight of the pyrolytic carbon. The carbon layer was mechanically removed at several points across the wafer, and the film thickness was determined using a Dektak 8 stylus profilometer.

### Micromechanical thermal analysis

The thermal analysis of the polymers was based on resonance frequency measurements of the pyrolytic carbon resonators. The resonance frequency was tracked using a commercial MSA-500 laser Doppler vibrometer from Polytec and an HF2LI lock-in amplifier from Zurich Instruments as previously described^32^. For temperature calibration, external heating was used, and the chip with the pyrolytic carbon resonators was placed on top of a Peltier element. The temperature was controlled by a thermocouple connected to the hot side of the Peltier element. The temperature was gradually increased, and the electrical resistance of the pyrolytic carbon resonators was recorded 10 min after changing the set value to allow for stabilization of the temperature. These measurements were used as calibration curves to determine the temperatures achieved by integrated resistive heating. For resistive heating, the pyrolytic carbon resonators were wire bonded to a printed circuit board (PCB), and a current was applied through the resonators (Fig. 3b, c). For thermal analysis, the applied current was stepwise increased, which resulted in an increase in the temperature of the resonators due to resistive heating of the carbon. Resistance measurements were performed immediately after setting the new temperature value. During the heating process, the resonance frequencies of the carbon resonators (blank and polymer coated) were monitored.

## Supplementary information

Supporting information
