# Supplementary material for: Pyrolytic carbon resonators for micromechanical thermal analysis
Source: Microsyst Nanoeng. 2019 Oct 21;5:58. doi: 10.1038/s41378-019-0094-x (PMC6803650; doi:10.1038/s41378-019-0094-x)
Supplement: Supplementary file 1 — Supporting information [file 41378_2019_94_MOESM1_ESM.docx]

**Supporting Information**

*for* **Pyrolytic carbon resonators for micromechanical thermal analysis**

Long Quang Nguyen^1,2,*^, Peter Emil Larsen^1,3^, Tom Larsen^4^, Sanjukta Bose-Goswami^1,3^, Luis Guillermo Villanueva^4^, Anja Boisen^1,3^ and Stephan Sylvest Keller^1,2^.

1 DNRF and Villum Fonden Center for Intelligent Drug Delivery and Sensing Using Microcontainers and Nanomechanics, IDUN, Technical University of Denmark, 2800 Kgs. Lyngby, Denmark

2 DTU Nanolab, Technical University of Denmark, 2800 Kgs. Lyngby, Denmark

3 DTU Health Tech, Technical University of Denmark, 2800 Kgs. Lyngby, Denmark

4 Advanced NEMS laboratory, Ecole Polytechnique Fédérale de Lausanne, 1015 Lausanne, Switzerland

**S1 – Resistance measurements for other pyrolysis conditions**

The resistance measurements for all the samples were performed with a probe station and a Parameter Analyzer HP4156A. Pyrolytic carbon string resonators at pyrolysis temperature 700^o^C and 1100^o^C were measured (Figure S1). Based on these results, the resistivity of pyrolytic carbon obtained at different pyrolysis temperatures were calculated by using the resistance and the dimensions of the resonators. The final resistivity was determined as the average value of 5 different values calculated for 5 different lengths of the resonators.

|  |  |
| --- | --- |

Figure S1: Resistance of pyrolytic carbon resonators fabricated at a) 700 ^o^C and b) 1100 ^o^C

**S2 – Resistivity calculation for different pyrolysis conditions**

Based on these results in S1, the resistivity of pyrolytic carbon obtained at different pyrolysis temperatures was calculated by using the resistance and the dimensions of the resonators. Based on the assumption that the cross-section of the resonator is uniform, the electrical resistivity *ρ* can be calculated by:

$\rho=R\frac{A}{l}$ = Rwt/l (S-1)

where R is the electrical resistance in Ω, l is the length of the resonator in m,A is the cross-sectional area of the resonator in m^2^, w is the width of the resonator in m and t is the thickness in m.

To calculate the cross-sectional area, the thickness of the pyrolytic carbon layer was measured with a stylus profilmeter while the width of the resonators was measured by optical microscope. The thickness of the pyrolytic carbon layer was measured at t=712±22 nm, t=640±7 nm, and 614±14 nm for 700ºC, 900ºC and 1100ºC, respectively. Figure S2 shows the measured width of pyrolytic carbon resonators fabricated at 700ºC, 900ºC and 1100ºC with different length of resonators. Considerable shrinkage of the pyrolytic carbon compared to the nominal width of the SU-8 precursor structures was observed. The decrease of thickness and width was more prominent for resonators fabricated with higher pyrolysis temperatures.

|  |  |
| --- | --- |
|  | |

Figure S2: Measured width of the pyrolytic carbon resonator with different lengths and nominal widths fabricated at a) 700ºC, b) 900ºC and c) 1100ºC.

With the measured width, thickness of the pyrolytic carbon resonators, the cross-sectional area of the resonators was calculated. Based on the equation (S-1) and the resistance of resonators measured above [S1], the resistivity of the resonators fabricated at 700ºC, 900ºC and 1100ºC was calculated at 39.4±6.8 Ω·cm, 3.8±0.1x10^-3^ Ω·cm, and 1.3±1.5x10^-3^ Ω·cm, respectively. The final resistivity was determined as the average value of 5 different values calculated for 5 different lengths of the resonators.

**S3 – Effect of readout laser power on resonance frequency**

Eventually, the laser of the vibrometer can be used as an external source to heat up the pyrolytic carbon resonators. The readout laser from the vibrometer was focused on the middle of the carbon resonator. The resistance of pyrolytic carbon resonators as well as the resonance frequency of pyrolytic carbon resonators were tracked for an increasing laser power. Using the TCR of pyrolytic carbon determined previously, the temperature of the resonator for a specific laser power could be estimated. Figure S3a shows the dependence of resonator resistance and resonance frequency on the laser power. The decrease of the resistance for increasing laser power confirm the behavior observed for external heating using the piezo crystal. Furthermore, the increase of the resonance frequency for increasing laser power support the conclusion of a negative value of the TEC for pyrolytic carbon. The contraction of the string due to local heating contributes to an increase in tensile stress and thereby an increase of the frequency. Figure S3b shows the relation between the laser power, the resonance frequency and the temperature.

| 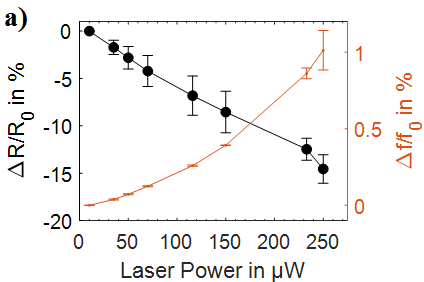 | 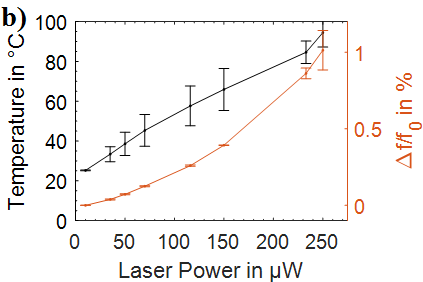 |
| --- | --- |

Figure S3: a) Dependence of resonators resistance and resonance frequency on the laser power and b) Relation between the laser power, the resonance frequency and the temperature

S4 – Resonator after T_m_ of PCL measurement

To prove that partial of PCL was reflow after Tm measurement, the SEM of the resonators after the experiment was obtained. Figure S4 shows the SEM image of the resonator after the T_m_ measurement.

| 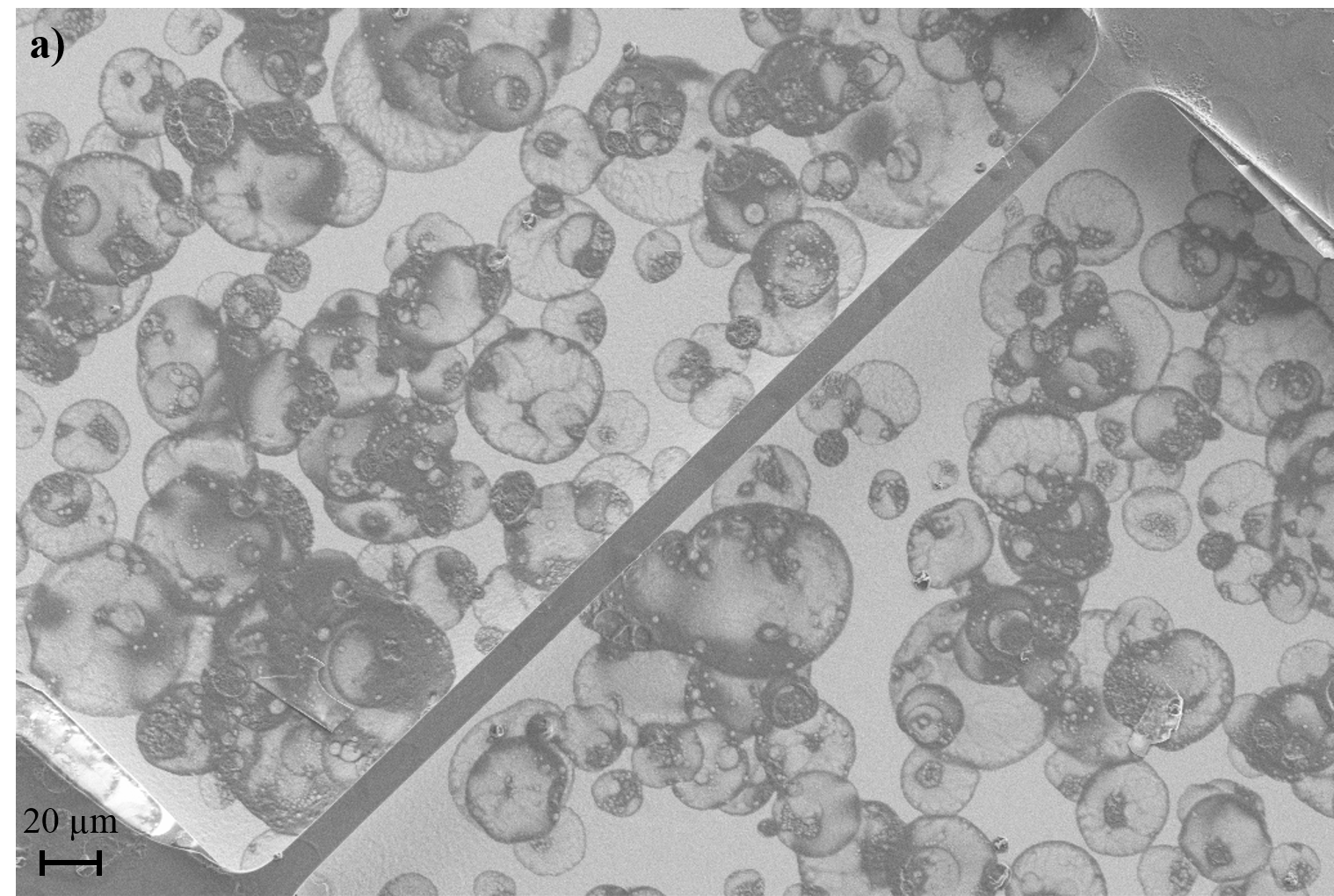 | 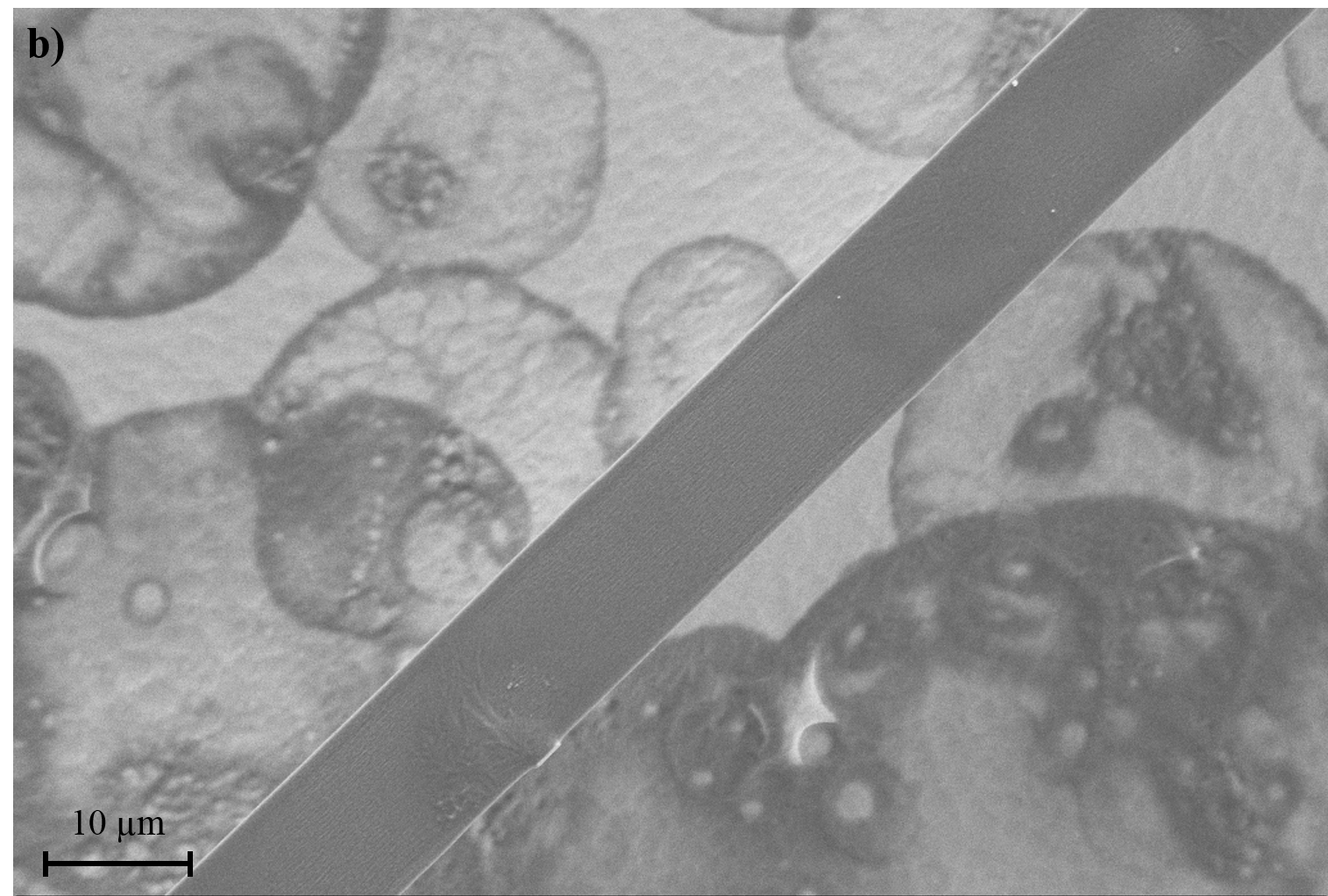 |
| --- | --- |

Figure S4: SEM images of the pyrolytic carbon resonator after T_m_ measurement of PCL.

The SEM shows that the PCL polymer actually reflows due to the melting phase of the polymer was achieved during the experiment. Furthermore, the resonance frequency of the resonator was measured after the integrated heating measurement. Figure S5 compares the resonance frequency of the new, before and after measurement of the pyrolytic carbon resonator. The results show the shift of resonance frequency due the change of PCL polymer mass distribution on the resonator.


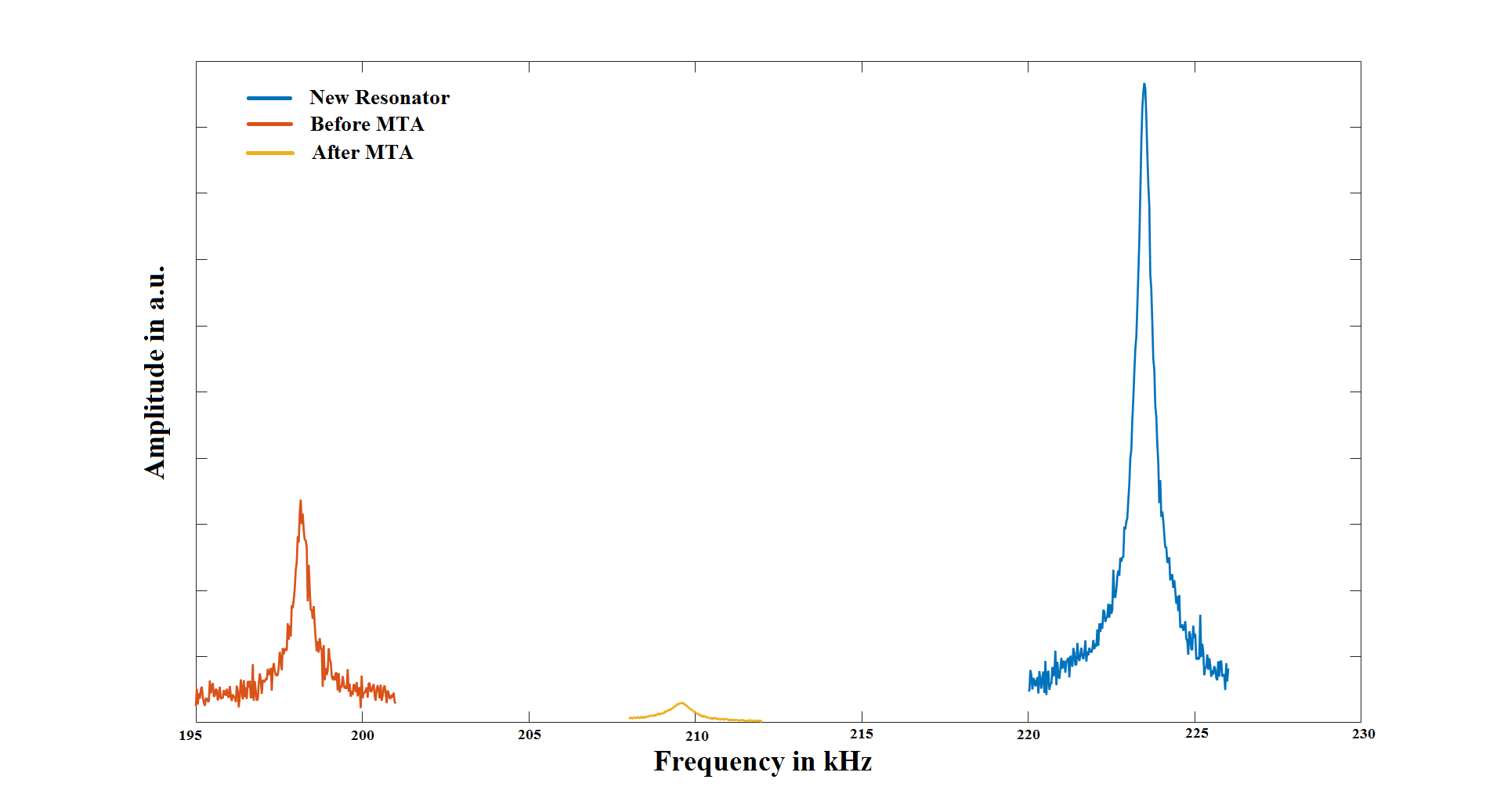


Figure S5: Resonance frequency of new, before and after MTA resonator.
